# Adding Humic Acids to Gelatin Hydrogels: A Way to Tune Gelation

**DOI:** 10.1021/acs.biomac.1c01398

**Published:** 2021-12-22

**Authors:** Virginia Venezia, Pietro Renato Avallone, Giuseppe Vitiello, Brigida Silvestri, Nino Grizzuti, Rossana Pasquino, Giuseppina Luciani

**Affiliations:** DICMaPI, Università degli Studi di Napoli Federico II, P.le Tecchio 80, 80125 Napoli, Italy

## Abstract

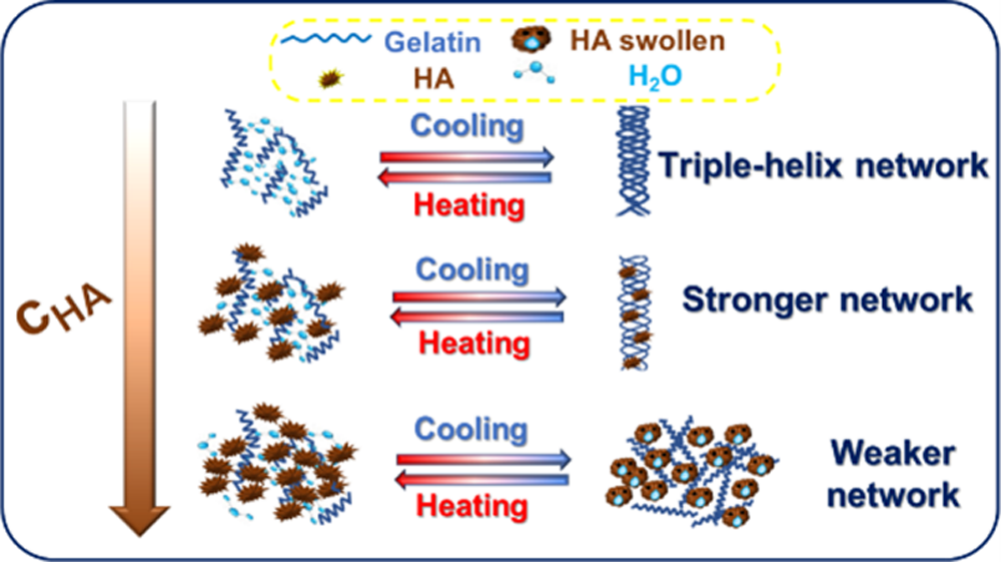

Exploring the chance to convert biowaste into a valuable resource, this study tests the potential role of humic acids (HA), a class of multifunctional compounds obtained by oxidative decomposition of biomass, as physical agents to improve gelatin’s mechanical and thermal properties. To this purpose, gelatin–HA aqueous samples were prepared at increasing HA content. HA/gelatin concentrations changed in the range 2.67–26.67 (wt/wt)%. Multiple techniques were employed to assess the influence of HA content on the gel properties and to unveil the underlying mechanisms. HAs increased gel strength up to a concentration of 13.33 (wt/wt)% and led to a weaker gel at higher concentrations. FT-IR and DSC results proved that HAs can establish noncovalent interactions through H-bonding with gelatin. Coagulation phenomena occur because of HA–gelatin interactions, and at concentrations greater than 13.33 (wt/wt)%, HAs established preferential bonds with water molecules, preventing them from coordinating with gelatin chains. These features were accompanied by a change in the secondary structure of gelatin, which lost the triple helix structure and exhibited an increase in the random coil conformation. Besides, higher HA weight content caused swelling phenomena due to HA water absorption, contributing to a weaker gel. The current findings may be useful to enable a better control of gelatin structures modified with composted biowaste, extending their exploitation for a large set of technological applications.

## Introduction

Gelatin, a natural peptide macromolecule obtained by partial hydrolysis of collagen, is one of the most employed polymers.^1,2^ Its large availability, low cost, biocompatibility, biodegradability, and poor antigenicity make it suitable for a wide number of applications in food as well as biomedical and pharmaceutical fields.^3−5^ Gelatin is easily soluble in water at temperatures above 30 °C, and a thermoreversible physical gel can be obtained by cooling gelatin aqueous solutions,^6−9^ as they undergo a sol–gel transition upon cooling, whose characteristics depend on various parameters (e.g., gelatin concentration, pH, etc.).^10−13^ Gelation of gelatin aqueous solutions has been widely studied in the literature.^14,15^ Several works reported the study of the time to onset of gelation under isothermal conditions,^16,17^ as well as the influence on the gelation temperature of a ramp rate applied to the solution.^10,13,18^

Gelatin properties can be improved by adding cosolutes, which interact through either chemical or physical junctions in a way to increase, on the one side, rheological and mechanical strength, or to confer, on the other side, peculiar properties, such as water resistance or thermal stability.^19−21^

Despite the great efficacy of the commonly used aldehyde cross-linkers (formaldehyde and glutaraldehyde), their toxicity poses health and safety issues and strongly limits their application, particularly in the biomedical field and food industry.^3,4^ Thus, there is a growing interest to find more sustainable and safe cross-linking choices based on natural moieties.^22^ Among these, polyphenols are known to interact with proteins, through physical and chemical conjugation, providing higher thermal stability as well as antioxidant features.^23^ For such reasons, polyphenols have been explored as additives for gelatin, yet with contrasting results. In some systems, gelatin benefits from their addition, exhibiting improved mechanical properties as well as higher thermal stability.^22,24^ In other cases, however, polyphenol addition results in lower tensile strength of gelatin films.^24^

Among bioavailable compounds, humic acids (HAs), the alkali-soluble fraction obtained from oxidative degradation of biomass in either natural or biorefinery processes, are intriguing moieties, with various functionalities in their backbone, including quinone, phenol, carboxyl, and hydroxyl groups, which confer them different properties, such as antioxidant, antibacterial, and anti-inflammatory activity. Thus, HAs are biowaste already available in nature in large quantities, as well as polyphenols, with the additional characteristic that HAs are more stable in terms of degradation.^25,26^ HAs also cost less than more common polysaccharides, such as pectin and agar,^27^ that are also widely used as additives for gelatin solutions, although they are obtained with a longer extraction. They hold, as such, a huge potential as a source for eco-sustainable materials. To this purpose, recent studies prove that HA blending with hydrogels leads to promising solutions, including biodegradable sorbents and delivery systems for a large number of applications.^28,29^

Despite the similarity of chemical functional groups with polyphenols, HAs have peculiar features.^30−33^ In fact, according to the currently accepted view, they are made of relatively low molecular weight compounds which are self-organized into supramolecular structures, held together by weak dispersive forces, such as van der Waals, π–π, and CH−π interactions.^34−36^ Furthermore, because of the noncovalent nature of stabilizing interactions, not only are these superstructures greatly affected by the chemistry of surrounding environment, including pH, cosolutes, and biological molecules, and behave as dynamic systems, but also they undergo self-restructuring in water.^33^ Because gel formation occurs in an aqueous environment, these features may have great influence on the gel behavior and cannot be disregarded. Therefore, the effect of HAs on gel properties of gelatin cannot be forecast by analogy based on the available studies on polyphenols–gelatin systems, which exhibit contrasting results.^22−24^ A dedicated investigation is required in order to provide a concrete chance for HA technological application, according to a waste-to-wealth approach. Prompted by this need, this study is focused on understanding the gel behavior and physicochemical properties of gelatin modified with HAs. To this purpose, gelatin from porcin skin (type A) was selected as the most exploited source for gelatin hydrogels,^37^ whereas Aldrich Humic Acid was selected as a model HA moiety.

Rheological properties have been investigated over a wide range of compositions (i.e., by tuning HA content, keeping fixed the gelatin concentration) to study the effect of HAs on the mechanical and thermal properties of the resulting hydrogel. In particular, gelation kinetics and transition temperatures have been investigated as a function of the HA concentration. Hydrogels have been characterized in terms of both the resulting strength and the chemical inter- and intramolecular interactions arising between gelatin and HAs. The physicochemical properties of gelatin–HA systems were assessed through X-ray diffraction (XRD), Fourier transform infrared spectroscopy (FT-IR), differential scanning calorimetry (DSC), scanning electron microscopy (SEM), and swelling kinetics to investigate the noncovalent interactions between HAs and gelatin and their role in conformational and rheological features.

## Materials and Methods

### Materials

**Figure 1:**
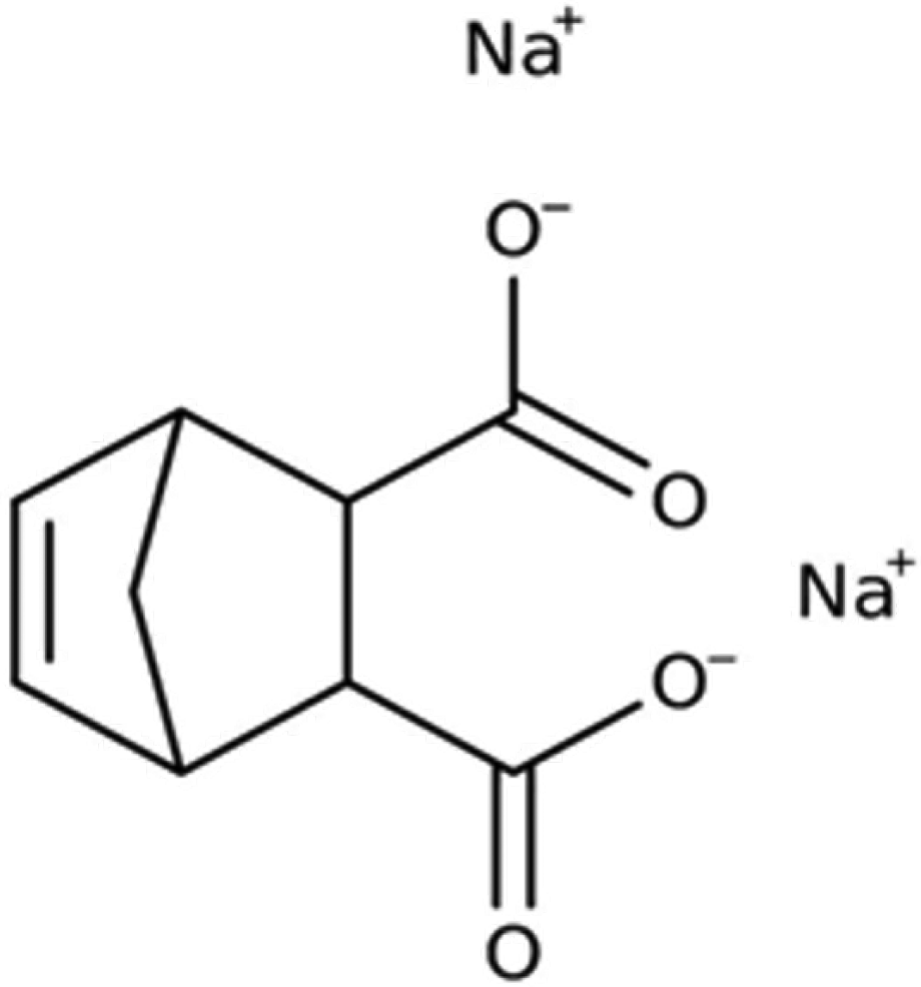
Two-dimensional chemical structure of HA sodium salt.

Gelatin from porcine skin (gel strength 300, type A) and humic acid sodium salt (HA), CAS Number 68 131-04-04, were obtained from Sigma-Aldrich (Milan, Italy) and used as received. Its chemical structure is reported in Figure 1. Elemental analysis of HA was reported elsewhere.^38,39^ Briefly, carbon, hydrogen, oxygen, nitrogen, and sulfur content were determined as 43.9, 3.5, 0.7, and <0.3%, respectively.

### Methods

#### Preparation of Neat Gelatin Solution

Gelatin powder was dissolved in bidistilled water at 5.7 wt % using a magnetic stirrer at 360 rpm and 60 °C for 30 min to guarantee complete dissolution. Gelatin concentration was chosen in order to work in a semidilute regime and avoid any precipitation during gel preparation. At the same time, this value is close to those reported in previous studies.^10,40^

#### Preparation of HA Solution

HA solution at 3 wt % was prepared by dissolving HA powder, added gradually, in bidistilled water. In particular, the solution was continuously stirred after each HA addition for 15 min and sonicated for 10 min. The obtained dispersion was kept for 2 days in order to separate the precipitate and the supernatant from the solution. The maximum water-soluble fraction of HA remaining in solution was 2 wt %.

#### Preparation of Gelatin–HA Solutions

Gelatin–HA solutions were prepared according to the following procedure. Gelatin solutions at different concentrations were prepared, and an appropriate volume was mixed with HA solution at 2 wt % to achieve the final gelatin concentration equal to 5.7 wt % and different HA composition as reported in Table 1. The obtained solutions were stirred at 360 rpm and 60 °C overnight, and the pH value was roughly 8. Samples were stored in glass bottles at room temperature.

**Table 1:** Composition of Gelatin–HA Samples

sample	HA composition (mg/ml)	% HA/gelatin (wt/wt)
gelatin–HA 1.6	1.6	2.67
gelatin–HA 8	8	13.33
gelatin–HA 16	16	26.67

### Dynamic Rheological Measurements

Rheological measurements were performed in a rotational stress-controlled rheometer (Discovery Hybrid Rheometer 2, TA Instruments, United States) equipped with a Peltier cell for temperature control and 40 mm diameter sandblasted parallel plates (thermal expansion coefficient of 0.957 μm/ °C). Tests were carried out by using a gap of 1 mm and a solvent trap to avoid evaporation at high temperature.

Dynamic temperature ramp tests (DTRTs) were performed by imposing a frequency of 10 rad/s and a deformation of 5% in a way to guarantee linear viscoelastic regime. They were used to evaluate the transition temperature of the solutions (reported in Table 1) in a temperature range between 60 and −5 °C, by imposing specific cooling and heating rates. Samples were loaded at 60 °C, cooled to −5 °C, and (after a waiting time of 300 s at −5 °C) heated again to 60 °C. The transition temperatures were defined as the minimum of derivative of  with respect to temperature.^10,41^ The transition temperature during cooling is indicated as *T*_sol–gel_, whereas the transition temperature during heating is indicated as *T*_gel–sol_.

Dynamic time sweep tests (DTSTs) were carried out in isothermal conditions to evaluate the gel time in a specific temperature range. The sample was loaded at 60 °C between the plates of the rheometer and cooled to the reference temperature test with an imposed cooling ramp of 10 °C/ min, with a frequency of 10 rad/s and a deformation of 5%. When the sample reached the reference temperature, the test started and the viscoelastic moduli were measured as a function of time. The gel time, *t*_gel_, was defined as the time at which the value of the storage modulus equals the value of the loss modulus.^16,42^

Dynamic frequency sweep tests were performed at 5 °C and with a linear strain of 5%. The viscoelastic moduli were measured in a frequency range between 100 and 0.1 rad/s.

#### Uniaxial Compression Tests

Uniaxial compression tests were performed on a rotational stress-controlled rheometer (MCR 702, Anton Paar, Austria) equipped with a Peltier cell for temperature control and 25 mm diameter parallel plates. Samples were taken out from the gel mold (after 16 h kept in the fridge) with a diameter of 7.5 mm and a height of 13 mm and placed on the Peltier unit on the center of the impacting plate. The gels were compressed with a crosshead speed of 1 mm/s at 5 °C. Six repeated experiments were performed for each HA concentration, and the stress–strain behavior of the gels was assessed.

The true stress (σ) and stretch ratio (λ) were evaluated by the measured normal force and reduced height, respectively, by following the procedure explained in Li et al.^43^

The stretch ratio was defined as
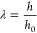
1where *h*_0_ and *h* are the initial gel height and its height after compression, respectively.

The true stress and strain were calculated as
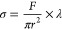
2
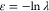
3

XRD, FT-IR, and TGA/DSC analyses were carried out on dried samples, which were obtained by an overnight thermal treatment at 60 °C to avoid any protein denaturation.

#### XRD Analysis

XRD patterns were tracked with a Malvern PANalytical diffractometer (Malvern, U.K.) with a nickel filter and Cu Kα radiation to investigate the crystalline phases of the gelatin protein and its structural changes in the solution due to the addition of HA. The relative intensity was recorded in the range of 2θ from 5^◦^ to 80^◦^.

#### FT-IR Spectroscopy

FT-IR spectroscopy was carried out on HA, gelatin and gelatin–HA samples through a Nicolet Instrument Nexus, Thermo Scientific, Waltham, MA, United States, equipped with a DTGS KBr (deuterated triglycine sulfate with potassium bromide windows) detector. FT-IR absorption spectra of all samples were recorded in the 4000–400 cm^–1^ range at a 2 cm^–1^ spectral resolution on pressed disks of powders previously diluted in KBr (1 wt %). The spectrum of each sample was corrected for that of blank KBr.

#### SEM analysis

SEM imaging was carried on HA, gelatin, and gelatin–HA samples using the following tool: FEI Ispect S; source, 6–12.5 kV; filament, tungsten equipped with an Everhart–Thornley detector (ETD).

Samples for SEM analysis were prepared following the procedure reported elsewhere,^44^ with some changes. Briefly, the samples were immersed in 1 mL pipet tips. Then, they were soaked in an aqueous solution of 1.6 wt % in glutaraldehyde and kept for 16 h, followed by washing with bidistilled water for 16h. All samples were dried and then sputter coated with gold before SEM analysis.

#### TGA/DSC Analysis

DSC measurements were carried out at low temperatures, using a TA Instrument simultaneous thermo-analyzer (DSC 2500 172.23.164.25) under nitrogen atmosphere (procedure: equilibrate at −120 °C, isothermal 2.0 min, ramp 10 °C/ min to 200 °C). Samples were hydrated with a defined amount of water (15 wt %) to verify the interaction with water through the evaluation of its temperature melting/crystallization. Furthermore, TGA/DSC measurements were recorded using a TA Instrument simultaneous thermo-analyzer (SDT Q600, New Castle, Delaware, United States). About 10 mg of each powder sample was put into a platinum pan and then heated to 600 °C, at a heating rate 10 °C/ min, under air atmosphere.

#### Swelling Analysis

The swelling kinetics of both gelatin and gelatin–HA hydrogels were determined following a well-known method reported in previous studies.^40,45^ Briefly, the investigated samples were dried at 50 °C, weighed, and then rehydrated in distilled water at room temperature. The samples were drained with filter paper in order to remove water in excess and weighed at 10, 20, 60, and 180 min. The swelling ratio was defined using eq 4:
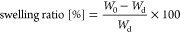
4in which *W*_0_ and *W*_d_ are the hydrated and the dried weight of the hydrogel, respectively.

## Results and Discussion

### Effect of HA on Viscoelasticity and Temperature Ramps

**Figure 2:**
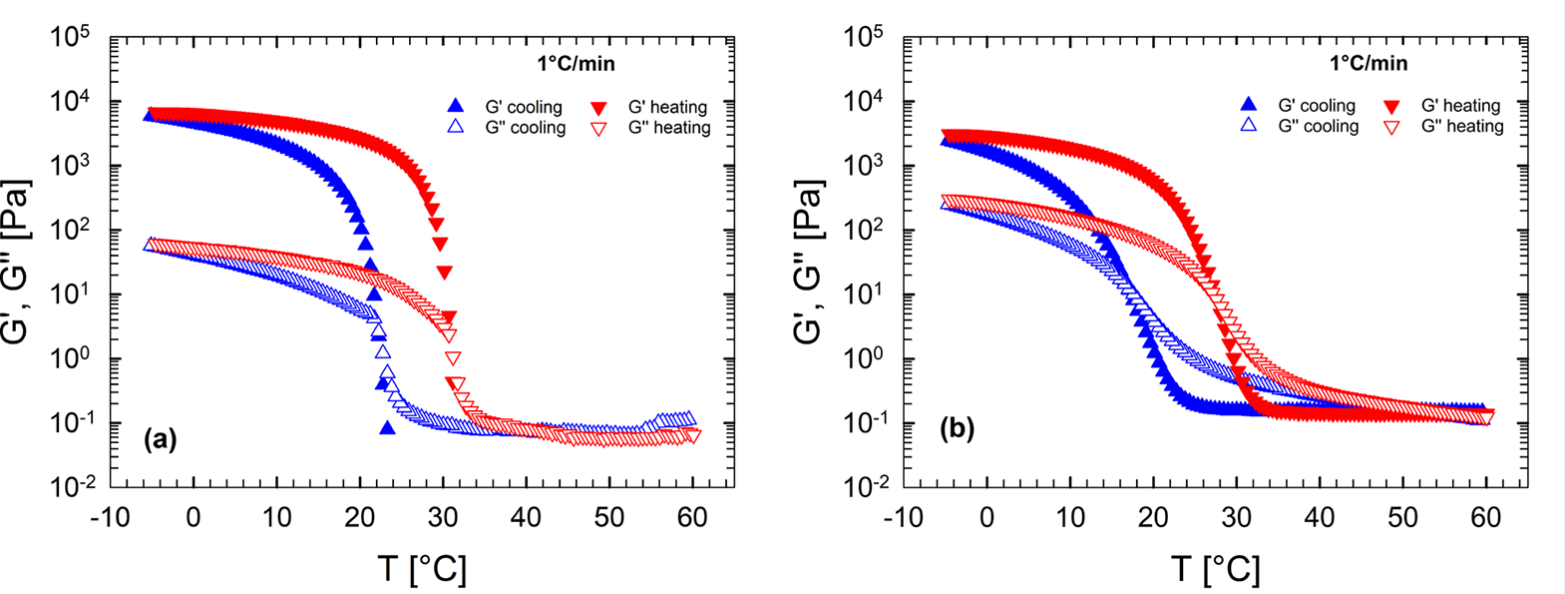
Viscoelastic moduli measured during cooling and heating ramps at 1 °C/min for (a) gelatin and (b) gelatin–HA 16.

Figure 2 shows the typical response of a dynamic temperature ramp test, both in cooling (blue curves) and in heating (red curves). The viscoelastic moduli are reported as a function of temperature for both solutions (Figure 2a, gelatin; Figure 2b, gelatin–HA 16). In both cases, at high temperature, the sample is a viscous liquid, as revealed by the very low viscoelastic moduli. Approaching a critical temperature, the viscoelastic moduli start to increase abruptly, indicating an incoming gelation process. At low temperature, below the critical temperature just discussed, the elastic modulus exceeds the viscous modulus by 2 orders of magnitude. This behavior indicates that a gel-like structure is formed. Then, when the temperature ramp is reversed, a melting process takes place, characterized by a steep decrease of the viscoelastic moduli. Finally, at high temperature, the moduli return to their initial values, indicating that the gelation is thermoreversible. The difference between the cooling and the heating ramps is indicative of a hysteresis, which depends on ramp rate.^10^ Various ramp rates were performed, and the transition temperatures were extracted, following Avallone et al.^10^ For more details, see Figure S1 in the Supporting Information.

**Figure 3:**
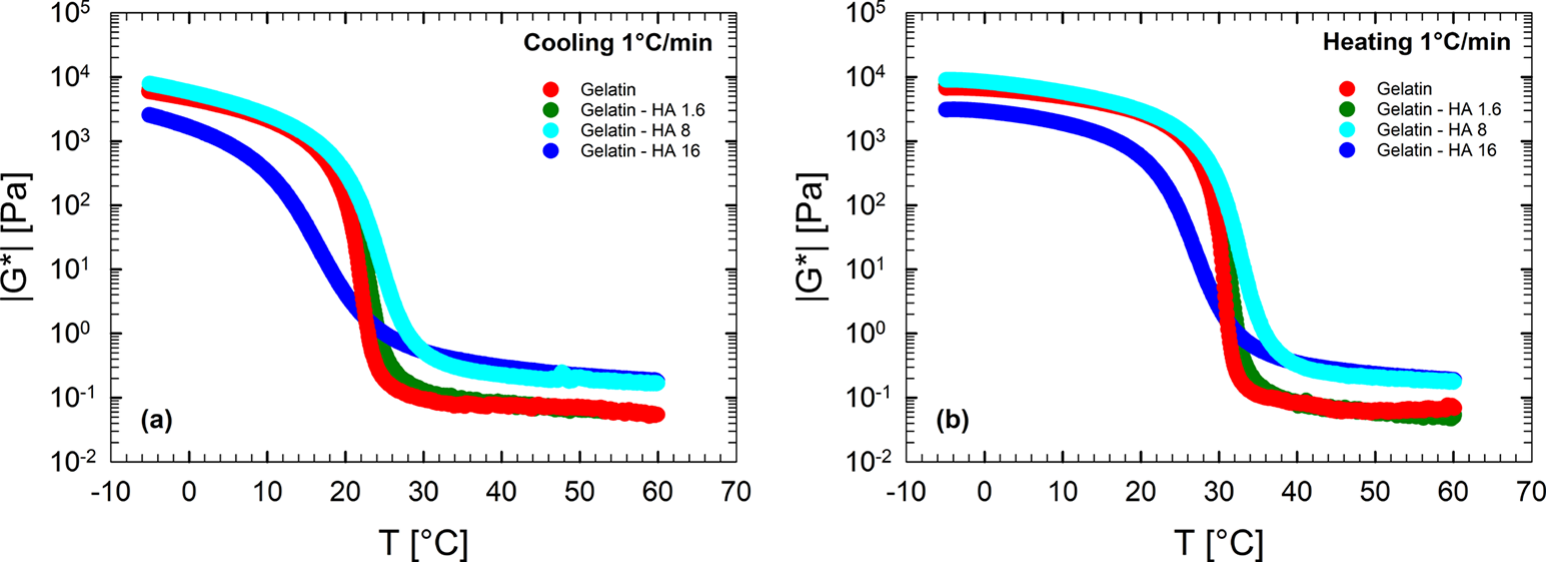
Complex modulus as a function of temperature at 1 °C/min and 10 rad/s for different concentrations of gelatin and humic acid aqueous solutions with (a) cooling and (b) heating ramp at 1 °C/min.

Panels a and b of Figure 3 report the complex modulus during cooling and heating at 1 °C/ min, respectively, for different systems containing various HA amounts. When HA content increases, the rheological curves shift nonmonotonically, suggesting the presence of a critical HA concentration, , for which the complex modulus exhibits the highest values, in both cooling and heating ramps.

**Figure 4:**
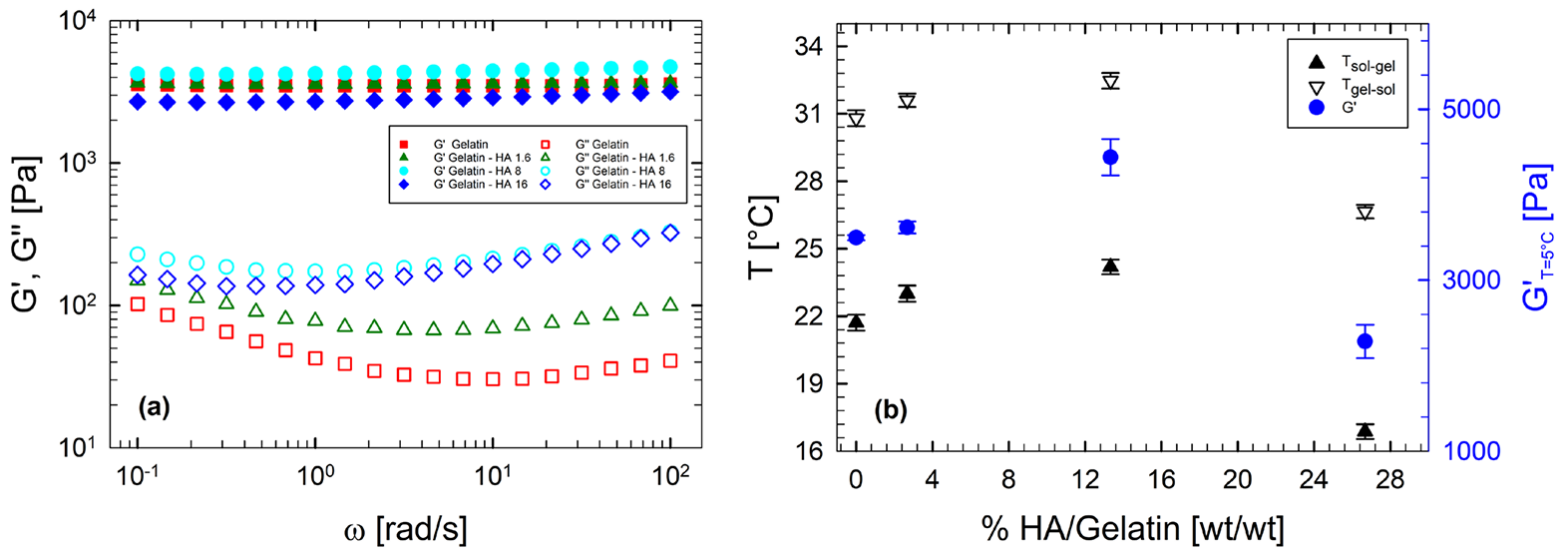
(a) Frequency sweep tests at 5 °C for samples with different HA concentrations. (b) Transition temperatures and elastic modulus at 5 °C and 10 rad/s as a function of HA content.

Figure 4a displays frequency sweep tests at 5 °C for various samples at different HA concentrations. The linear rheological response is typical of an elastic network, with *G*′ higher than *G*″ and independent of frequency, regardless of the HA content considered. Figure 4b reports the transition temperatures in cooling (*T*_sol–gel_, filled triangles) and in heating (*T*_gel–sol_, empty triangles), measured at 1 °C/ min together with the elastic modulus at 5 °C and 10 rad/s as a function of HA content. The elasticity of the resulting gel (right axis in Figure 4b) changes in a nonmonotonic way according to HA amount. A low content (up to 13.33 (wt/wt)%) increases the viscoelasticity of the resulting gel, whereas a higher HA amount builds a “softer” gel, characterized by a larger value of the viscous modulus and a lower value of the elastic modulus. In other words, when HAs exceed a specific amount, their presence reduces the “distance” between *G*′ and *G*″. Even the transition temperatures show the same nonmonotonic trend as a function of the HA content.

Notably, HA addition to gelatin up to a concentration of 13.33 (wt/wt)% has both *G*′ and *G*″ increased, resulting in |G*| values greater than that of the water/gelatin system. These features evidence that the mechanical and thermal stabilities of the gel improve because of the presence of HA. Notably, it must be argued that up to a concentration limit, HA has a beneficial effect on the gelation process, promoting formation of a tighter network.

### Effect of HA on Gelation Kinetics

**Figure 5:**
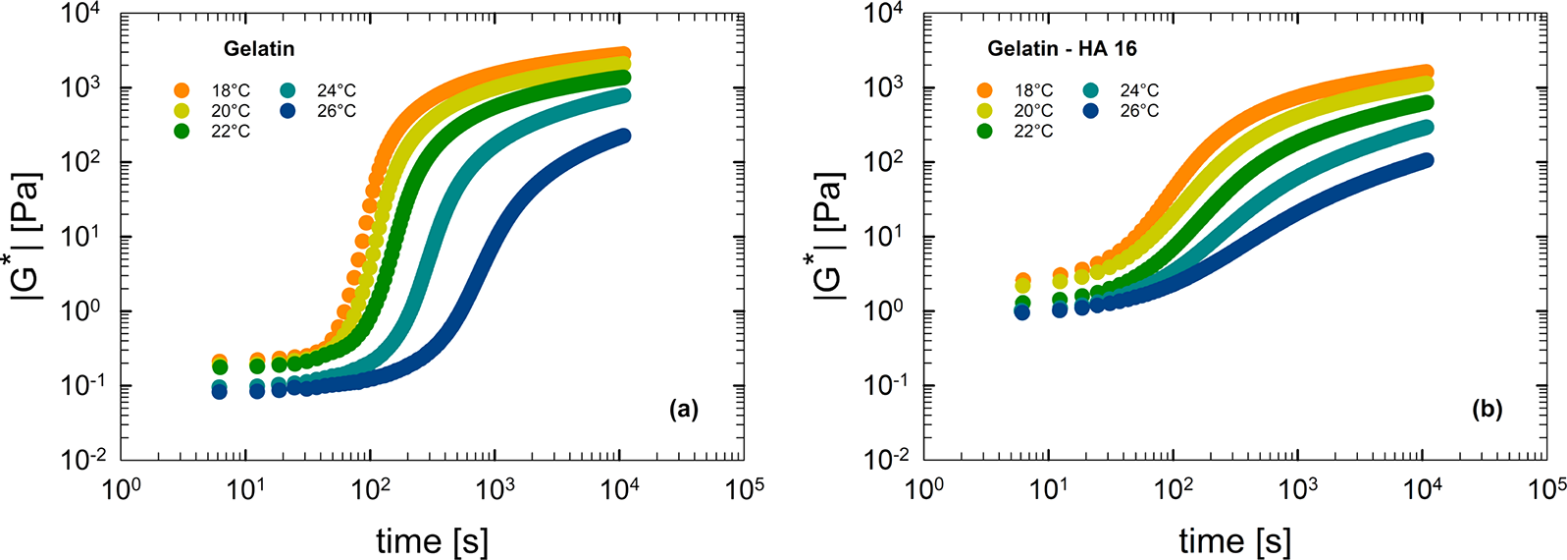
Complex modulus as a function of time for different temperatures for (a) gelatin and (b) gelatin–HA 16 samples.

Panels a and b of Figure 5 show the isothermal kinetics for gelatin and gelatin–HA 16 samples, respectively. In particular, the complex modulus as a function of time is reported at different temperatures. The gelation process can be followed with time by the transient increase of the complex modulus, being faster at lower temperatures. Moreover, by comparing the data plotted in Figure 5a,b at the same temperature (same curve color), it is evident that, at short times, the value of |G*| is higher for the gelatin–HA 16 sample. This means that HAs affect the rheological properties, because they increase the viscosity of the final solution. Conversely, at long times, a weaker gel (characterized by a lower value of |G*|) is obtained for the gelatin–HA 16 sample.

**Figure 6:**
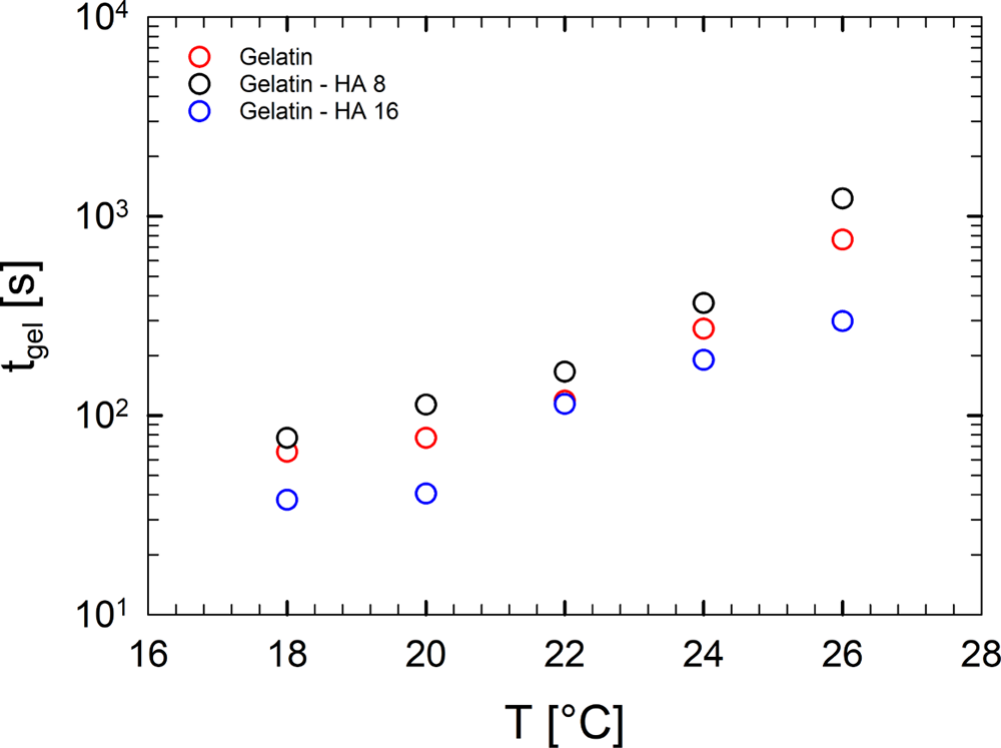
Temperature dependence of the gel time.

Figure 6 displays the temperature dependence of the gel time for gelatin, gelatin–HA 8, and gelatin–HA 16 samples, highlighting that HA slightly affects *t*_gel_ because the time necessary to obtain a gel is marginally influenced.

In order to investigate the reasons for this rheological behavior, a detailed physicochemical characterization was carried out.

### Effect of HA on Compression Tests

**Figure 7:**
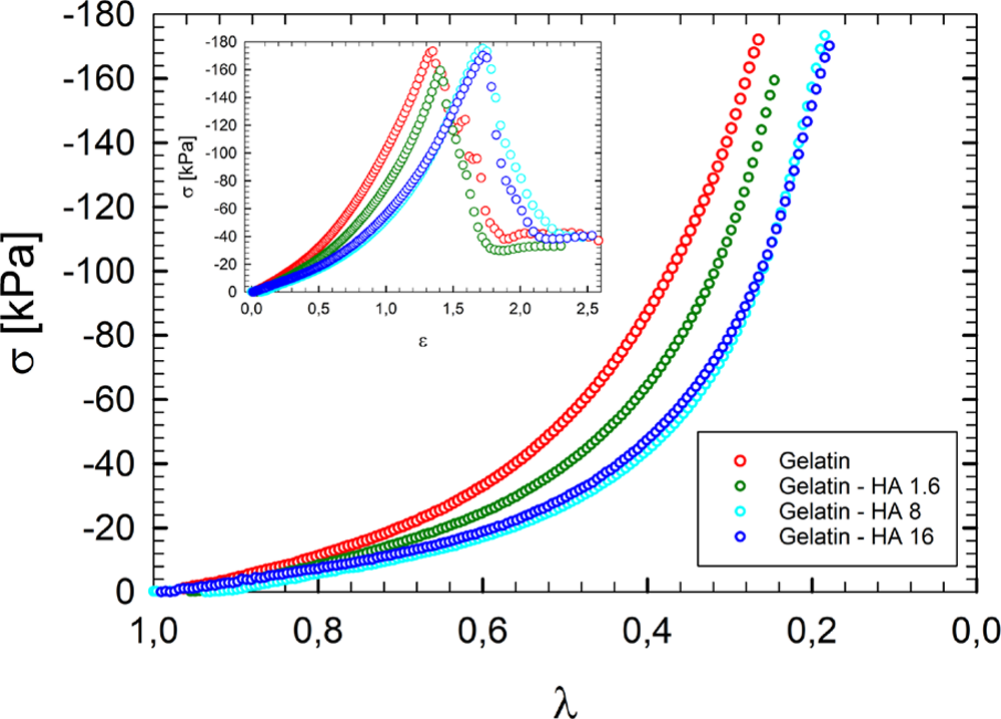
Uniaxial compression experiments; true stress plotted as a function of the stretch ratio for the samples reported in Table 1. The inset reports stress–strain curves.

The compression responses of gelatin gels with and without HA are shown in Figure 7a. Samples display nonlinear elasticity and strain hardening at large stretch ratio. The value of deformation at break increases on increasing HA concentration, showing a tighter network that must be broken under the effect of the uniaxial compression in the presence of HA. The stress at break instead shows a nonmonotonic behavior with HA content, although it is not significant. Such features can be easily detected by the inset in Figure 7a, which reports also the abrupt decrease of true stresses after breaking.

### XRD Analysis

**Figure 8:**
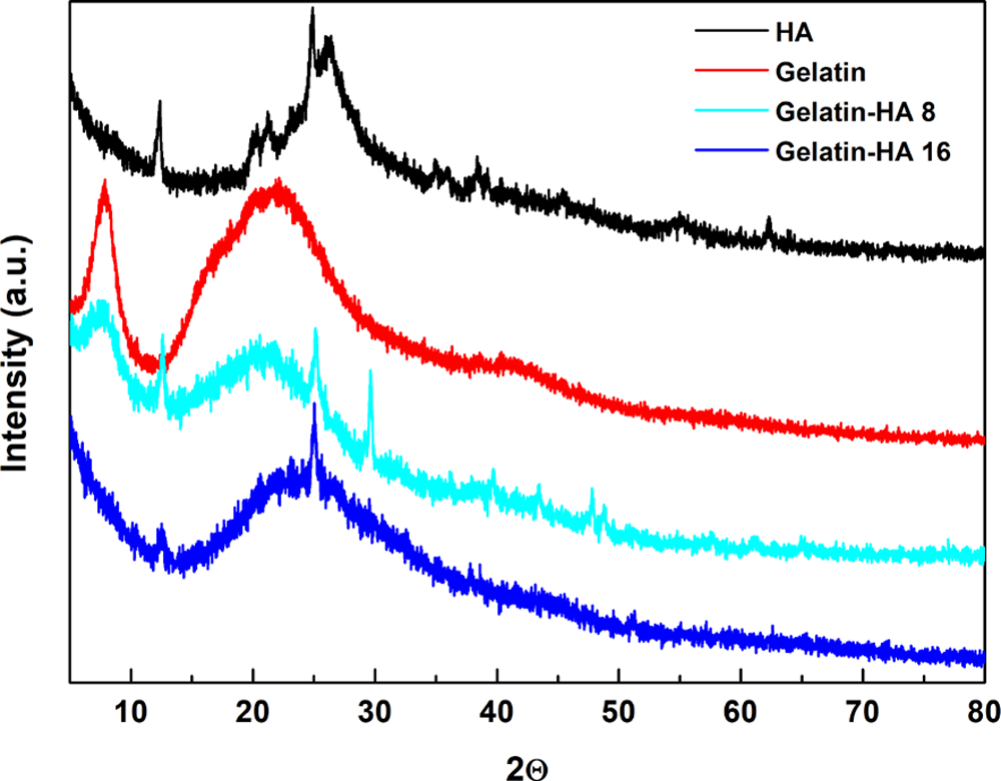
XRD patterns of gelatin, HA, gelatin–HA 8, and gelatin–HA 16.

Figure 8 shows XRD profiles of gelatin, HA, gelatin–HA 16 and gelatin–HA 8 samples. XRD patterns of bare gelatin and HA are reported in the same panel for comparison. The HA XRD profile, displayed in Figure 8 with black curve, exhibits a broad halo, revealing its amorphous structure except for some weak diffraction peaks related to the presence of some inorganic materials, typical of clay soil. The gelatin XRD pattern shows a sharp and a broad peak at 2θ of 8° and 20°, respectively, typical of a partially crystalline gelatin structure.^46^ Notably, the former is assigned to the ordered triple helical crystalline structure, whereas the latter confirms the presence of α-helix in the protein.^47^ The peak at 2θ of 8° is strongly reduced in the XRD profile of the gelatin–HA 16 sample, indicating a decrease of the content of triple helices in the sample and, thus, a change in the secondary structure of gelatin due to its mixture with HA.^46^ Instead, the gelatin–HA 8 XRD pattern shows that the peak at 2θ of 8° is only slightly reduced, suggesting that in this case the conformation of the protein is better preserved after mixing with HA. These results are in agreement with the rheological findings, which highlighted a weaker gel for the gelatin–HA 16 sample. In fact, at high amounts, HAs influence the protein secondary structure, preventing gelatin chains from organizing into triple helix domains and causing the formation of more disordered organization.

### FT-IR Spectroscopy

**Figure 9:**
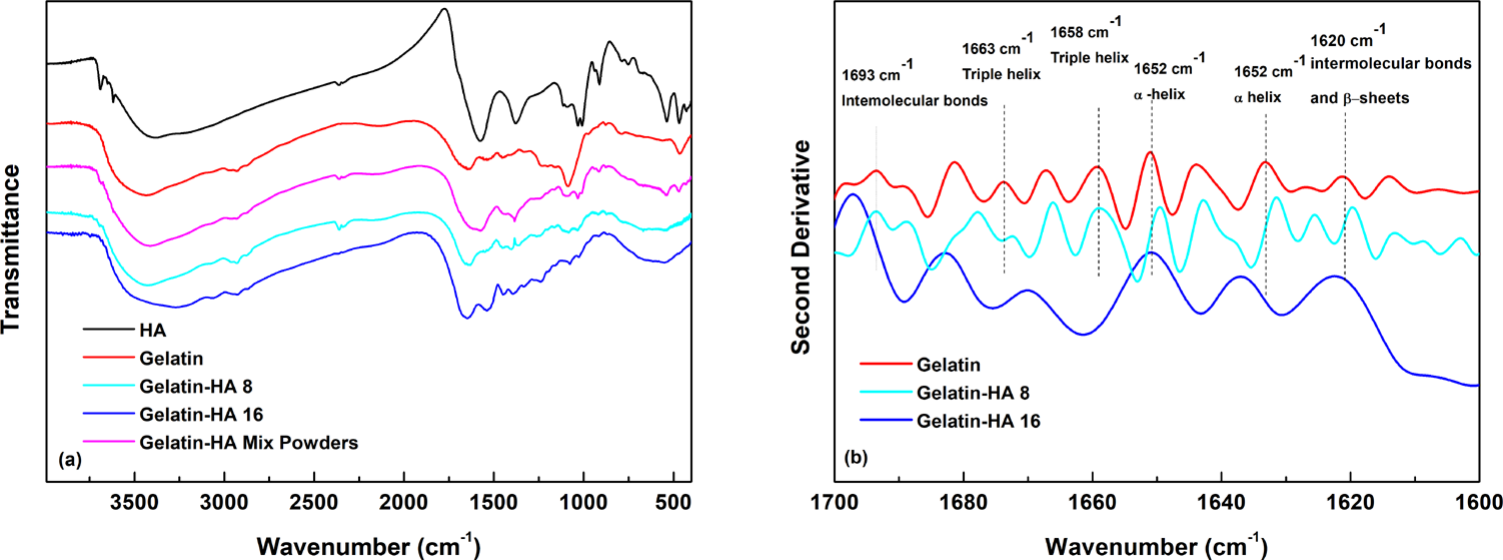
(a) FT-IR spectra and (b) second derivative spectra.

FT-IR analysis was carried out to identify functional groups of gelatin, HA, gelatin–HA, and gelatin–HA mix powders and to assess if any change occurred because of gelatin–HA interactions (Figure 9a). Tables 2 and 3 list the band assignment in the FT-IR spectra of gelatin and HA, respectively.^48,49^ The FT-IR spectra of gelatin and gelatin–HA 8 are similar. This evidence is due to the high amount of gelatin in the sample, which covers the HA FT-IR characteristic bands. On the other hand, in the FT-IR spectrum of gelatin–HA 16 the band in the range 3500–3400 cm^–1^, assigned to N–H and O–H stretching vibration modes, shifts toward lower wavenumbers (3270 cm^–1^).^50,51^ This can be attributed to the H-bond interactions between carbonyl groups of HA and H atoms in gelatin residues, further confirming the interaction between HA and gelatin (observed in the TGA analysis, Figure S2). With a closer look, in the FT-IR spectrum of gelatin–HA 16, the amide I band changes its shape, moving toward lower wavenumbers, suggesting that the peaks, related to unordered structures, become more prevalent, indicating protein restructuring due to the mixture with HA. Furthermore, the bands at 1653, 1540, and 1400 cm^–1^, respectively, related to ν_C=O_ and ν_NH_ stretching vibrations in amide I and to ν_NH_, ν_C–N_, ν_C–C_, and  stretching modes in amide II, grow more intense in the FT-IR spectrum in the gelatin–HA 16 sample.^50,51^ This might be partly due to the presence of HA, whose peculiar bands occur in the same ranges. These modifications are a further proof of a conformational change of the gelatin secondary structure.

**Table 2:** Infrared Spectral Characteristics of Gelatin

region	wavenumber (cm^–1^)	functional groups
amide A	3430	ν_NH_, ν_OH_
amide B	3060	ν_NH_
amide B	2930	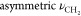
amide I	1650	ν_C=O_, ν_NH_
amide II	1540	ν_NH_, ν_C–N_, ν_C–C_
amide II	1450	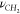
amide II	1410	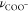
amide II	1330	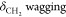
amide III	1235	δ_C–N_, δ_NH_
amide III	1080	ν_C–O_

**Table 3:** Assignment of FT-IR Bands of HA

wavenumber (cm^–1^)	functional groups
3690	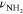
3390	phenolic −OH hydroxyl groups
2925	aliphatic C–H bands
1575	antisymmetric ν_COO^–^_ of carboxyl salt
1380	symmetric ν_COO^–^_ of carboxyl salt
1100	ν_CO_ (phenolic), ν_OH_ (aliphatic)
1040	ν_C–N_
1005	ν_CO_
910	out-of-phase δ_CH_ (aromatic)

More insight can be obtained by calculating the second derivative of FT-IR spectra in the range of 1600–1700 cm^–1^, which is reported in Figure 9b. This enabled resolving amide I band into six main peaks at 1693, 1663, 1658, 1652, 1630, and 1620 cm^–1^, related to the presence of intermolecular associations: triple helix (1658 and 1663 cm^–1^), single α-helix (1652 cm^–1^), β-sheets (1620 cm^–1^), β-turns (1693 cm^–1^,) and random coil (1630 cm^–1^).^52,53^

In the second derivative of gelatin–HA 16, the peak of the unordered structure is prevalent, whereas those related to the triple helix are no longer evident. These features suggest that the protein undergoes a conformational change due to its interaction with HA. This is also confirmed by the shift toward lower wavenumbers of the peaks at 1693 and 1620 cm^–1^, related to intermolecular associations in the gelatin matrix. Changes in the second derivative are more evident in the sample with the highest HA concentration (Figure 9b), confirming the key role of HA in modifying the protein structure.

Notably, high concentration of HA leads to a random coil organization, because the protein chains are not able to establish triple helix domains, typical of an ordered structure. This is also confirmed by XRD spectra that show a more unordered structure of gelatin due to the presence of HA in the matrix. In contrast, the second derivative of gelatin–HA 8 suggests that the helical structures are much better recovered, indicating that HAs act through hydrogen bond interactions with gelatin, also improving the elastic properties of the resulting gel, as shown in Figure 4.

HAs are mixtures of relatively low molecular weight compounds, characterized by a great variety of functional groups, including quinone, phenol, carboxyl, and hydroxyl moieties.^35,54^ HAs are organized in sopramolecular soft and permeable clusters, exposing hydrophilic groups, whereas hydrophobic species are shielded in the interior.^55,56^ These chemical features make HAs highly reactive with proteins.^57^ Accordingly, they can interact with gelatin through noncovalent interaction, such as hydrophobic and electrostatic interactions, as well as H-bonds. Notably, H-bond interactions should be established between carbonyl groups of HA and hydroxyl groups of hydroxyproline and proline residues as well as amino moieties of glycine, which are the most abundant amino acids in gelatin chains.^58^ This hypothesis is supported by previous studies, reporting interactions between gelatin and polyphenols, which bear similar functional groups as HA.^50,59^ Indeed, electrostatic interactions between HA and gelatin should be negligible, because the pH value of HA–gelatin solutions is roughly 8 (close to the gelatin isoelectric point) and both moieties are expected to be negatively charged. Thus, HAs improve elastic properties of the final gels if their concentration does not exceed 13.33 (wt/wt)% dry gelatin. In particular, this kind of interaction accounts for gel formation in the investigated samples, as evidenced by the marked dissolution of the gel in urea solution, which is effective in breaking hydrogen bonds.^44^ More specifically, FT-IR spectra and XRD patterns (Figures 8 and 9a) clearly evidence that HAs affect gelatin structure. Notably, the addition of higher HA concentrations hinders the protein structuring into the triple helix conformation, probably because water molecules are constrained by interactions with HA moieties, preventing H-bonds between gelatin and water.^60,61^ In this way, they may interfere with the hydrogen bonding among three α-chains required to form the triple helix structure.

### SEM Analysis

**Figure 10:**
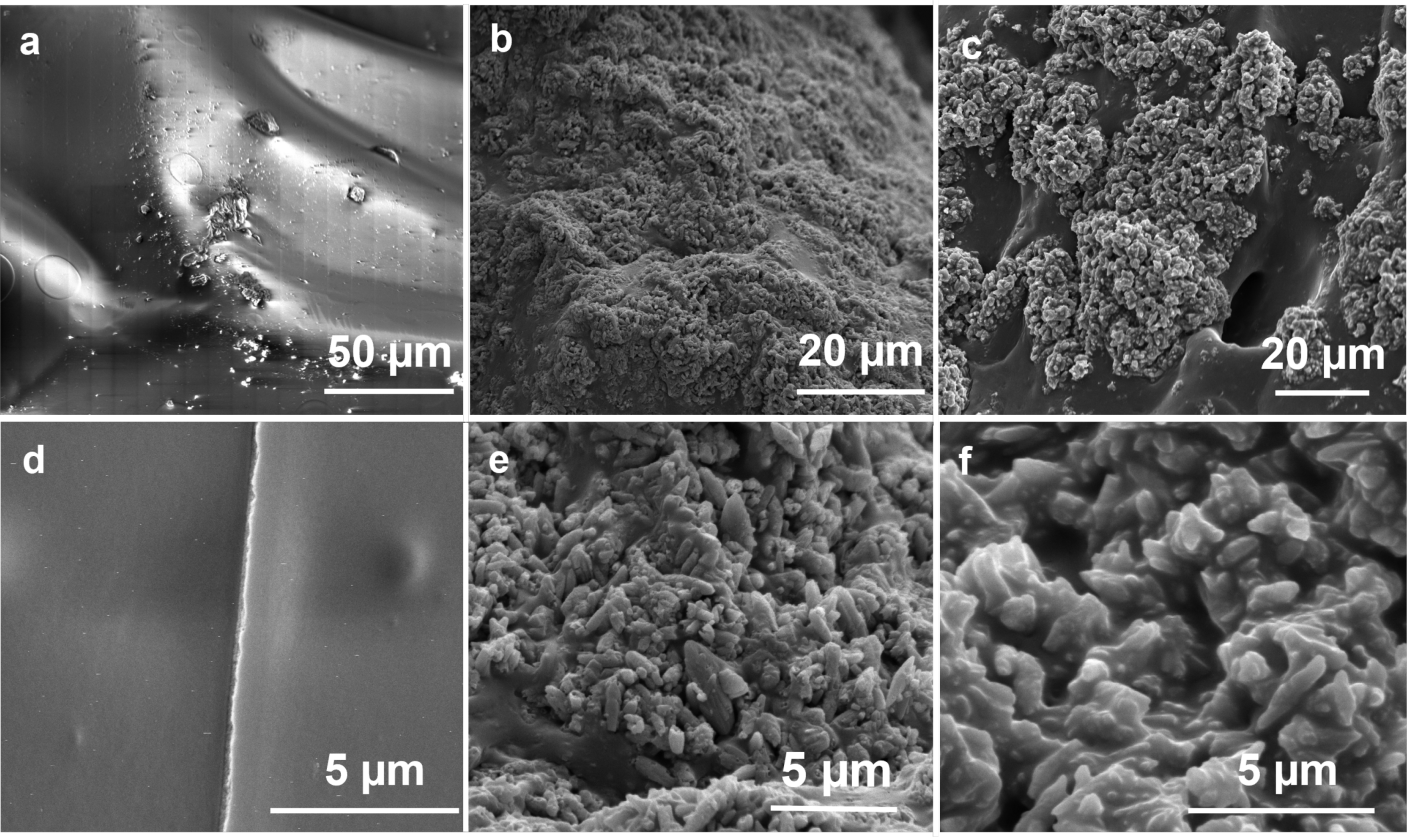
SEM Pictures of gelatin (a and d), gelatin–HA 8 (b and e), and gelatin–HA 16 (c and f).

Figure 10 shows SEM pictures of gelatin and gelatin–HA surfaces. Bare gelatin exhibits a smooth surface (Figure 10a,d), whereas the introduction of HA determines a significant change in gelatin structure, producing a rougher surface (Figure 10b,c,e,f) due to the presence of submicrometric particle aggregates. These results suggest that the interaction between gelatin and HAs induces a partial coagulation of the protein, preventing the formation of the ordered structure.^40,62^ This phenomenon occurs at a larger extent in the presence of higher HA content in the samples. In fact, the gelatin–HA 16 sample shows the most heterogeneous structure, with large aggregates and large voids. Observed aggregates might be produced by the precipitation phenomena caused by the interaction between HAs and gelatin. A similar behavior was already observed by the addition of polyphenols, which caused gelatin coagulation. Precipitation could prevent gelatin chains from reverting to triple helix structure and causes a decrease in the gel strength.^63,64^ These findings are in accordance with FT-IR and XRD results, which evidence that an excessive amount of HAs in the gel causes the loss of triple helix organization in the gelatin network.

### Low-Temperature DSC

A number of studies report the key role played by water in the network formation of gelatin.^16,17,65^ At the same time, HAs show great affinity toward water because of their hydrophilic groups.^33,66^ Therefore, HAs and gelatin might compete for interactions with water molecules.

**Figure 11:**
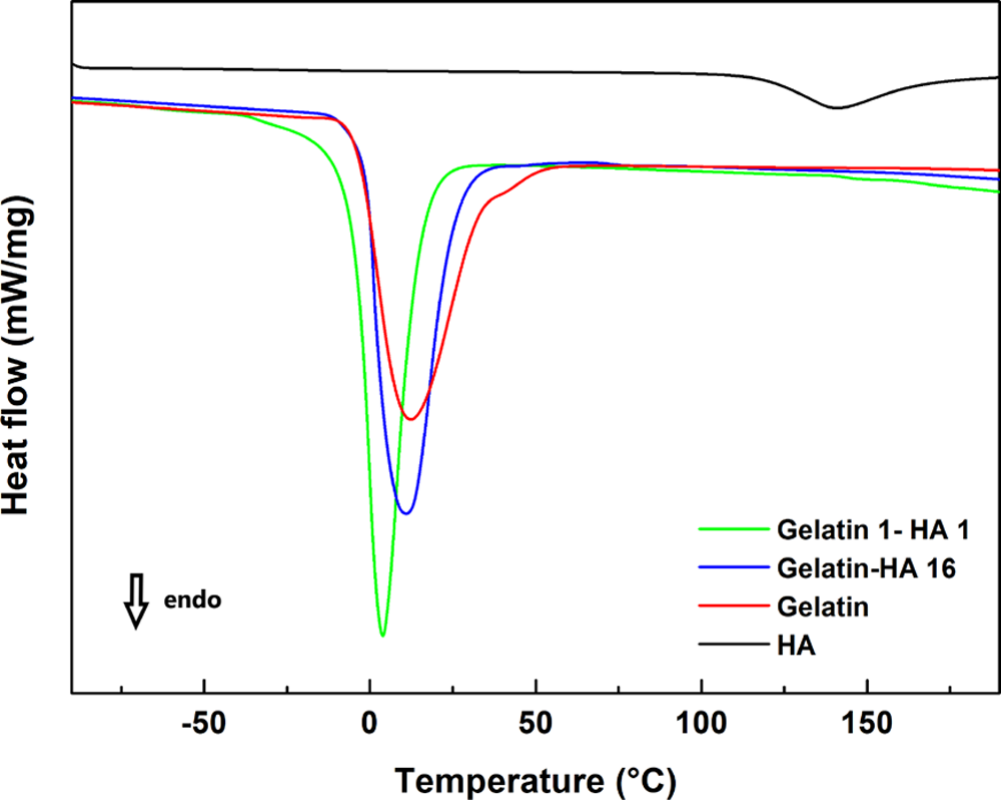
DSC curves of sol 1 (red curve, pure gelatin), sol 2 (blue curve), sol 3 (green curve), and HA (black curve).

In order to investigate if any selective affinity occurs between water and HA or gelatin, respectively, low temperature DSC analysis was carried out on gelatin films containing different amounts of HA (20 wt % and 50 wt %). Figure 11 shows the DSC curves, evidencing an endothermic peak at about 0 °C for the bare gelatin (red curve), related to the melting point of crystallized water.^65^ Instead, this effect is not present in the DSC curve of bare HA (black curve), suggesting that water molecules are constrained by intimate interactions with HA moieties, which prevent them from crystallization.^66^ Water affinity to HA must be larger than to bare gelatin, whose DSC profile shows an evident ice melting peak, suggesting the presence of a relevant amount of crystallized water. To highlight a significant change in crystallization water by varying HA concentration, a sample in which HA concentration is equal to that of gelatin (green curve) has been prepared. In this aspect, it is possible to observe that the peak area decreases and peak temperatures shift to lower values by increasing HA content in the samples, confirming that upon increasing HA amount, the content of crystallizable water decreases. The great ability of HA to attract and interact with water molecules should enable formation of more protein–protein junctions, increasing the network tightness and gel viscosity. Similar results were obtained by introducing sugar molecules into gelatin solutions.^22^ On this basis, it can be inferred that in gelatin–HA samples, HAs preferentially interact with water molecules and “sequester” them, preventing their coordination with gelatin chains. It is known that water plays a key role in stabilizing the triple helix structure and the arising gel network, because it is able to act as a bridging agent between gelatin chains through H-bond interactions.^60,67^

Elastic properties show a nonmonotonic trend with the HA content in the samples. Both elastic and viscous moduli, *G*′ and *G*′′, increase up to an HA critical concentration of 13.33 (wt/wt)% dry gelatin, whereas *G*′ decreases at a greater HA content. This behavior is in accordance with other studies reporting the rheological and physicochemical features of gelatin hydrogels including polyphenols.^40,68,69^ However, the amount of HA that can be added to gelatin solution without impairing its elastic properties is significantly larger than the usually used polyphenols content. This can represent an intriguing feature for technological applications in view of antioxidant or antimicrobial properties of HAs. Indeed, the best-performing composition of the system gelatin–HA is equivalent to that of other studies, reporting the use of Galla Chinensis as an additive for gelatin.^69^ Nevertheless, compared to natural polyphenols, HA have the great advantage of being more stable toward oxidation phenomena.

In order to explain the non-monotonic trend of gelatin rheological properties with HA content, we deduce that if HAs do not exceed a critical HA concentration (), the presence of tight physical interactions between HA and gelatin and the ability to attract water molecules improve gel strength. However, high HA content more likely produces coagulation of gelatin and the further increase of viscosity prevents interchain interactions because of the inhibition of gelatin molecules from approaching each other required to achieve an ordered organization. At the same time, water absorption by HAs can produce relevant swelling phenomena;^70,71^ consequently, protein chains interact less through interchain hydrogen bonds, thus producing a weaker gel, as confirmed by the decrease of the elastic modulus, previously shown in Figure 4.

### Swelling Analysis

**Figure 12:**
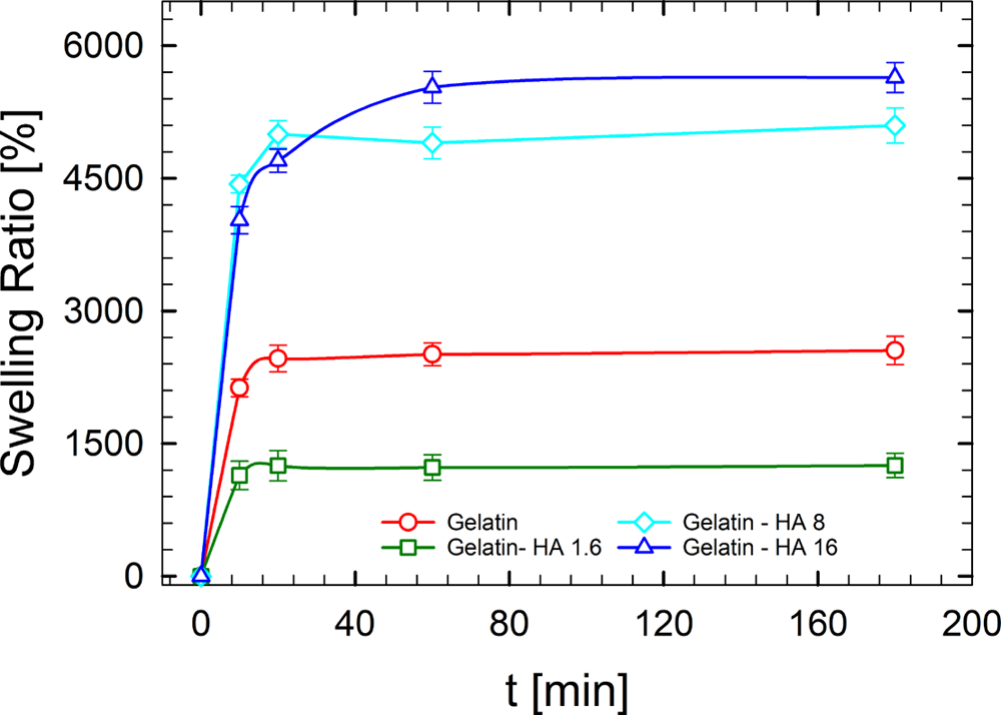
Swelling kinetics of gelatin hydrogels with and without HA at different concentrations.

Figure 12 shows swelling kinetics of neat gelatin and gelatin–HA samples. The swelling ratios of bare gelatin and gelatin–HA 1.6 samples steadily increased, reaching a constant value soon after 20 min, whereas gelatin–HA 8 and gelatin–HA 16 specimens achieved constant weight after 60 min, evidencing slower swelling kinetics with higher water absorption. In fact, constant swelling ratios of the control, gelatin–HA 1.6, gelatin–HA 8, and gelatin–HA 16, were 2500%, 1200%, 5000% and 5500%, respectively. Therefore, HA addition to gelatin resulted in a nonmonotonic trend of swelling ratio, with gelatin–HA 1.6 showing the lowest value among investigated samples, revealing a decreased ability to absorb water. Furthermore, measured values for gelatin–HA 8 and gelatin–HA 16 specimens are much higher than those reported in the literature for polyphenol containing gelatin.^40^ The obtained results evidence that gelatin–HA 8 samples combine relevant water absorption with improved elastic properties.

The swelling ratio is usually influenced by cross-linking density as well as hydrophilicity.^40,72^ From the analysis of obtained results it can be inferred that at low HA concentration, physical junctions between HAs and gelatin result in a lower ability to absorb water. On the other hand, HAs evidence a marked hydrophilic behavior, proven by low-temperature DSC analysis as well as relevant swelling phenomena in water, usually with slow kinetics.^33^ These features must prevail at high content, leading to a marked improved water uptake, but with slower swelling rates as shown in Figure 12.

## Conclusions

The incorporation of HAs into the gelatin network affects the viscoelastic properties as well as the thermal stability of the resulting gel. Rheological characterization and in-depth physicochemical investigation of gelatin samples at various HA concentrations demonstrated that HAs affect the final gel structure through additional H-bonds. In addition, gelation kinetics is only slightly influenced by HA concentration, proving that humic acids marginally affect the time needed to obtain a gel with respect to a water/gelatin solution. Nevertheless, a nonmonotonic behavior in the elastic, swelling, and thermal properties of the final gels was found, by varying HA content in solution.

At low HA concentrations (up to 13.33 (wt/wt)%), HAs improve both elastic and thermal properties of the obtained gels because of H-bonds’ intimate interactions with gelatin chains, as also suggested by FT-IR results.

Physical interactions between HA and gelatin also reduce the water absorption ability of the final gel up to HA concentration of 2.67 (wt/wt)%.

Low-temperature DSC analysis evidences that HAs have higher water affinity than gelatin. Therefore, at higher HA concentrations, elastic properties and thermal stability decrease because of the HA-driven swelling phenomena. This implies that the interchain interactions with the gelatin matrix are, as such, inhibited. On the other hand, the marked hydrophilic features of HAs result in swelling ratio increase.

Furthermore, XRD and the second derivative of FT-IR spectra proved that a higher HA content in solution leads to a prevailing random coil gelatin organization, because the protein chains are not able to establish wide triple helix domains.

Our results shed light on the key aspects that might contribute to a more conscious management of gelatin properties, in the presence of bioavailable moieties, such as HAs, contributing to the expansion of their repurposing in a wide range of applications.

## Supporting Information Available

*T*_sol–gel_ and *T*_gel–sol_ as functions of the imposed ramp rate for various ternary systems (HA, gelatin, and water); TGA curves of the investigated samples (gelatin, HA, gelatin–HA 0.8, gelatin–HA 1.6, and gelatin–HA mix powders) (PDF)
