# Supplementary material for: Adding Humic Acids to Gelatin Hydrogels: A Way to Tune Gelation
Source: Biomacromolecules. 2021 Dec 22;23(1):443–53. doi: 10.1021/acs.biomac.1c01398 (PMC8753605; doi:10.1021/acs.biomac.1c01398)
Supplement: Supplementary file 1 — bm1c01398_si_001.pdf [file bm1c01398_si_001.pdf]

Adding humic acids to gelatin hydrogels: a way to tune gelation

Virginia Venezia,[†,‡] Pietro Renato Avallone,[†,‡] Giuseppe Vitiello,[†] Brigida Silvestri,[†] Nino Grizzuti,[†] Rossana Pasquino,[*,†] and Giuseppina Luciani[*,†]

†*DICMaPI, Università degli Studi di Napoli Federico II, P.le Tecchio 80, 80125 Napoli, Italy*

‡*Contributed equally to this work*

E-mail: r.pasquino@unina.it; luciani@unina.it

Supporting Information Available

Figure S1 reports $T_{sol-gel}$ and $T_{gel-sol}$ as a function of the imposed ramp rate. As expected, the $T_{sol-gel}$ has a larger dependence on the ramp rate with respect to $T_{gel-sol}$. Both $T_{sol-gel}$ and $T_{gel-sol}$ move to lower temperatures at higher HA concentration (2.67 (wt/wt)%). On the other hand, samples with low quantity of HA (13.33 (wt/wt)% and 26.67 (wt/wt)%) show higher transition temperatures compared to the systems Gelatin and Gelatin-HA 1.6.

TGA curves of investigated samples (Figure S2) clearly show lower degradation kinetics than bare gelatin.[1,2] This does not occur in a physical mixture of powdered HA and gelatin (magenta curve), which evidences a TGA curve comparable to that of bare gelatin. These results suggest that interaction must be established between HA and gelatin, due to their intimate mixing and accounting for improved thermal stability.[3]

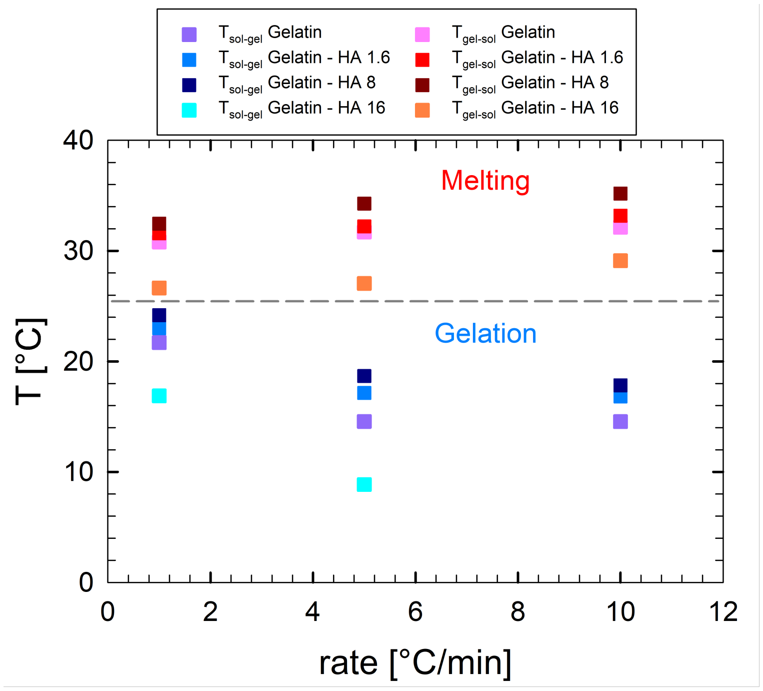

Figure S1: $T_{sol-gel}$ and $T_{gel-sol}$ as functions of the imposed ramp rate for various ternary systems (HA, gelatin and water - see legend for details). The horizontal dashed line marks the two transitions.

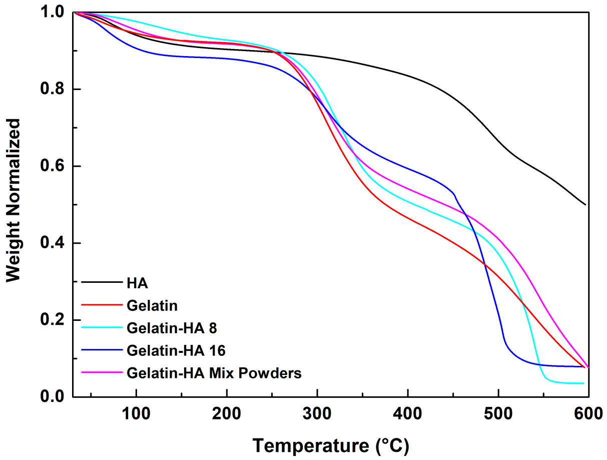

Figure S2: TGA curves of Gelatin, HA, Gelatin-HA 0.8, Gelatin-HA 1.6 and Gelatin-HA Mix Powders.

References

(1) Zhao, Y.; Sun, Z. Effects of gelatin-polyphenol and gelatin–genipin cross-linking on the structure of gelatin hydrogels. *Int. J. Food Prop.* **2017**, *20*, 2822–2832.

(2) Lei, J.; Li, X.; Wang, S.; Yuan, L.; Ge, L.; Li, D.; Mu, C. Facile fabrication of biocompatible gelatin-based self-healing hydrogels. *ACS Appl. Polym. Mater.* **2019**, *1*, 1350–1358.

(3) Russo, P.; Venezia, V.; Tescione, F.; Avossa, J.; Luciani, G.; Silvestri, B.; Costantini, A. Improving Interaction at Polymer–Filler Interface: The Efficacy of Wrinkle Texture. *Nanomaterials* **2020**, *10*, 208.
